# Continuous Flumazenil Infusion and Time to Consciousness Recovery in Benzodiazepine Poisoning: A Retrospective Cohort Study

**DOI:** 10.3390/jcm14175983

**Published:** 2025-08-24

**Authors:** Jisu Kim, Soo Hyun Kim, Seung Pill Choi, Jong Ho Zhu, Sung Wook Kim, Mi Kyong Kwon, Jae Hun Oh

**Affiliations:** Department of Emergency, Eunpyeong St. Mary’s Hospital, College of Medicine, The Catholic University of Korea, Seoul 03312, Republic of Korea; 6fold@naver.com (J.K.); unidgirl@catholic.ac.kr (S.H.K.); emvic98@catholic.ac.kr (S.P.C.); kingmonst2r@gmail.com (J.H.Z.); mdkaptain@naver.com (S.W.K.); artient@hanmail.net (M.K.K.)

**Keywords:** benzodiazepines, flumazenil, poisoning, time factors, adverse effects

## Abstract

**Background:** Benzodiazepine poisoning is a frequent cause of emergency department (ED) visits, often related to suicide attempts. Flumazenil is the only specific antidote, but its continuous infusion protocol remains controversial because of its uncertain outcome benefits and increased risk of adverse events. This study aimed to evaluate the effect of continuous flumazenil infusion on the time to recovery of consciousness and secondary outcomes in patients with benzodiazepine poisoning stratified by hospitalization status. **Methods:** A retrospective cohort study was conducted at a tertiary hospital in Seoul, Korea, including adults treated for benzodiazepine poisoning in the ED between April 2019 and March 2024. The primary outcome being the time from arrival at the ED to regaining consciousness. Multivariate regression identified independent predictors of delayed recovery. **Results:** Among the 370 patients, 52.4% were hospitalized. Flumazenil infusion was administered in 46.8% of the patients, more often in hospitalized patients. In this group, flumazenil infusion significantly reduced the median time to regain consciousness (13.7 vs. 19.4 h, *p* = 0.006) but did not affect the overall hospital stay. In nonhospitalized patients, flumazenil infusion did not shorten the awakening time or prolong the ED stay. Adverse events, mainly agitation, were more frequent with flumazenil infusion. **Conclusions:** Continuous infusion of flumazenil accelerates the recovery of consciousness only in hospitalized patients who are severely affected by benzodiazepine poisoning but with increased adverse events and no reduction in hospital stay. Individualized patient selection and evidence-based protocols are needed for optimal and safe antidote use.

## 1. Introduction

Benzodiazepine poisoning remains a significant clinical challenge in emergency medicine worldwide [1,2,3,4]. Benzodiazepines are among the most commonly prescribed psychotropic medications and are widely used for their anxiolytic, hypnotic, muscle relaxant, and anticonvulsant properties. However, their therapeutic ubiquity also contributes to their frequent involvement in intentional and unintentional overdoses. Benzodiazepine overdose is a leading cause of drug-related emergency department (ED) visits and is often associated with deliberate self-harm or suicide attempts [1,5]. The clinical presentation of benzodiazepine poisoning ranges from mild drowsiness to profound coma and respiratory depression, necessitating prompt recognition and management to prevent morbidity and mortality.

The management of benzodiazepine toxicity is primarily supportive, with a focus on airway protection, respiratory support, and hemodynamic stabilization. Flumazenil, a competitive benzodiazepine receptor antagonist, is the only specific antidote available for reversing benzodiazepine-induced central nervous system depression [6]. While flumazenil can rapidly restore consciousness in selected patients, its use remains controversial due to the risk of precipitating withdrawal seizures, particularly in individuals with chronic benzodiazepine use, co-ingestion of proconvulsant agents, or underlying seizure disorders. Consequently, clinical guidelines recommend cautious and selective administration of flumazenil, often reserving its use for cases of isolated benzodiazepine overdose without contraindications.

Despite its potential to expedite recovery of consciousness, the real-world impact of flumazenil—especially when it is administered as a continuous infusion—on clinical outcomes such as time to awakening, length of hospital stay, and adverse event rates remains inadequately characterized. Most literature focuses on single-dose administration, with limited data on the efficacy and safety of continuous infusion protocols in diverse patient populations [7,8,9,10]. Furthermore, the relationships between flumazenil use and key outcomes may differ between patients requiring hospitalization and those managed in the ED without admission. Understanding these nuances is crucial for optimizing treatment strategies and improving patient care in cases of benzodiazepine poisoning. Flumazenil acts as a competitive antagonist at the benzodiazepine binding site of the gamma-aminobutyric acid (GABA)-A receptor, but experimental data suggest it may also exert modulatory effects on other receptor systems, which could influence clinical outcomes. Its elimination half-life (approximately 40–80 min) is substantially shorter than that of many benzodiazepines, predisposing patients to re-sedation once plasma flumazenil concentrations decline while long-acting benzodiazepines remain active. This pharmacokinetic mismatch is a central reason why bolus-only regimens may fail to sustain consciousness. Continuous infusion maintains steadier plasma levels and theoretically prevents re-sedation, whereas repeated bolus administration can result in fluctuating arousal. The present study investigates the continuous infusion approach in the real-world ED setting [11].

This study aims to address these gaps by investigating the associations between the continuous infusion of flumazenil and the time to recovery of consciousness in patients presenting with benzodiazepine poisoning. By utilizing a large, retrospective cohort from a tertiary urban hospital in Seoul, Korea, we sought to evaluate the effectiveness and safety of flumazenil infusion in both hospitalized and nonhospitalized patients. We further examined the impact of flumazenil on secondary outcomes, including ED and hospital length of stay and the incidence of adverse events. By providing detailed, stratified analyses, our findings can inform clinical decision-making and contribute to the development of evidence-based protocols for the management of benzodiazepine overdose.

## 2. Materials and Methods

### 2.1. Study Design

This retrospective and observational study was conducted at Eunpyeong St. Mary’s Hospital, an 830-bed teaching hospital located in an urban area of Seoul, Korea. The ED of this hospital accommodates approximately 50,000 patients annually. The study protocol was reviewed and approved by the institutional review board (Approval Code PC24RISI0211, dated on 19 December 2024). Owing to the retrospective nature of the study, the requirement for informed consent was waived.

### 2.2. Study Population

**Figure 1:**
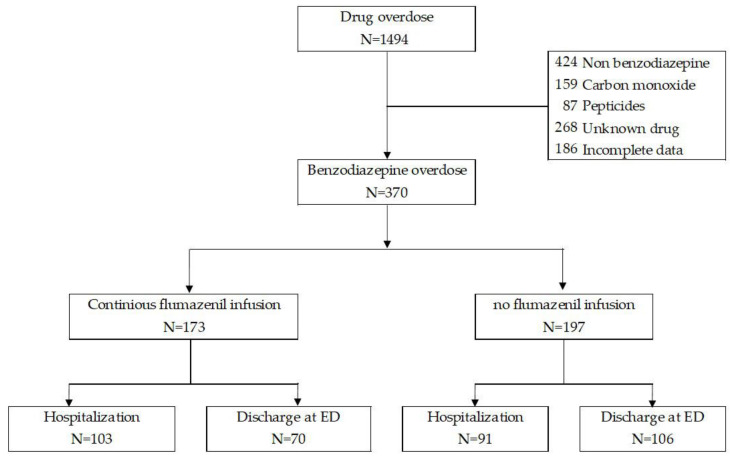
Selection of the Study Population with Benzodiazepine Poisoning. ED, emergency department.

All adult patients, defined as individuals older than 18 years who visited the ED for any medical problem between April 2019 and March 2024, were screened for eligibility. In South Korea, the cause of each ED admission is immediately reported to the National Emergency Department Information System (NEDIS), which records detailed information for cases of deliberate self-harm, including the specific method of the suicide attempt. The NEDIS records categorize the methods as poisoning, cutting or piercing, suffocation, hanging or choking, drowning or near drowning, using fire or heat, falling or jumping from a great height, other methods, or unknown. All patients who visited the ED after a suicide attempt were included in the NEDIS records. To minimize missing data, we supplemented the NEDIS data with additional information obtained through postmanagement, outpatient clinic follow-up, specialist consultations, and thorough review of medical records. Only patients who presented with objective evidence of a suicide attempt, and among them, only those with benzodiazepine poisoning, were included in the final analysis. The process of patient selection and inclusion is illustrated in Figure 1.

### 2.3. Data Collection

For each patient included in the study, demographic and clinical data were obtained from medical records. The variables collected included age, sex, occupation, and living condition (classified as living with or without family). The amount of benzodiazepine ingested was recorded, and to standardize this measurement across different types and brands, the dose was converted to the defined daily dose (DDD), which is a standardized unit representing the average maintenance dose per day for a drug used for its main indication in adults [12]. Additional data collected included the presence of co-ingestion of other drugs or alcohol and any previous psychiatric history. The laboratory data obtained at the time of ED admission included blood gas analysis. At the time of arrival at the emergency department, vital signs and level of consciousness were assessed, with the level of consciousness expressed using the Glasgow Coma Scale (GCS) prior to any flumazenil administration. “Recovery of consciousness” was defined as attainment of GCS 15 or the ability to maintain airway patency and engage in coherent verbal interaction.

### 2.4. Overdose and Treatment

For all cases of benzodiazepine overdose, we further documented whether there was co-ingestion of other drugs or alcohol, whether flumazenil continuous infusion was administered, and, if so, the duration and total dose of flumazenil used. The time elapsed from ingestion to ED arrival, as well as the time from ED arrival to recovery of consciousness, were carefully recorded. The use of continuous flumazenil infusion, duration, and total dose were considered potentially influential variables in the clinical course and outcome of benzodiazepine poisoning. Activated charcoal administration was defined as delivery of 25–50 g orally or via nasogastric tube within two hours of ingestion, unless contraindicated (e.g., unsecured airway without intubation).

### 2.5. Outcome Variables

The primary outcome of this study was the time from ED arrival to recovery of consciousness, which was defined as the restoration of a clinically appropriate level of awareness and responsiveness and determined based on medical records indicating that the patient was able to have an interview, reflecting a clinically relevant and objectively measurable reversal of central nervous system depression with implications for airway safety. This variable was analyzed by grouping patients into quartiles based on time to recovery, with the range and distribution of values including means and standard deviations specified to allow for detailed interpretation and reproducibility. The secondary outcomes included the occurrence of critical events, such as seizures, aggressiveness or tachycardia; the length of stay in the ED; in-hospital mortality; the total length of hospital stay (from admission to discharge); and whether the patient was admitted to the hospital or discharged from the ED. Tachycardia was defined as any episode of heart rate exceeding 100 beats per minute sustained for more than 10 min, as recorded by continuous cardiac monitoring during the emergency department stay. Seizures were identified based on documented clinical observations of generalized tonic–clonic or focal motor activity, or the administration of anticonvulsant medication as recorded in the medical records. Other adverse events, such as agitation and pneumonia, were also documented through medical record review, with agitation defined as the presence of clinically significant restlessness or aggressiveness requiring medical intervention. All clinical events were retrospectively verified through detailed chart review by trained investigators to ensure consistency and accuracy.

### 2.6. Statistical Analysis

Categorical variables are presented as frequencies and percentages, and comparisons between groups were performed using the chi-square test or Fisher’s exact test, as appropriate. The distributions of continuous variables were assessed for normality by visual inspection and the Shapiro–Wilk test. Normally distributed data are expressed as the means and standard deviations and were compared using Student’s *t* test, whereas nonnormally distributed data are expressed as medians and interquartile ranges and were compared using the Mann–Whitney U test. To assess independent predictors of time to recovery of consciousness, multivariate linear regression analysis was performed. All variables with a significance level less than 0.1 in the univariate analysis were included in the multivariate linear regression model. All the statistical analyses were performed using SPSS software, version 23.0 (IBM Corp., Armonk, NY, USA), and values of *p* less than 0.05 were considered statistically significant for all the comparisons.

## 3. Results

### 3.1. Patient Characteristics

The study cohort comprised 370 patients with benzodiazepine poisoning, of whom 194 (52.4%) required hospitalization and 176 (47.6%) were discharged from the emergency department (Figure 1). Male patients represented 28.1% of the total cohort, with no significant difference between the hospitalized and nonhospitalized groups (28.4% vs. 27.8%, *p* = 1.000). The median age was 46.0 years, with hospitalized patients having a slightly greater median age (49.0 years) than nonhospitalized patients (45.0 years), although this difference was not statistically significant (*p* = 0.158).

Hospitalized patients demonstrated significantly lower Glasgow Coma Scale (GCS) scores upon arrival at the emergency department than nonhospitalized patients did (median 11.0 vs. 13.0, *p* < 0.001). Additionally, hospitalized patients had higher benzodiazepine ingestion doses measured in defined daily dose units (13.1 vs. 10.0, *p* = 0.004). Living with family was significantly more common among hospitalized patients (58.2% vs. 35.8%, *p* < 0.001) (Table 1).

### 3.2. Clinical Outcomes with Flumazenil Infusion

Flumazenil continuous infusion was administered to 173 patients (46.8% of the total cohort), with significantly greater usage in hospitalized patients than in nonhospitalized patients (53.1% vs. 39.8%, *p* = 0.014). The median flumazenil dose was substantially greater in hospitalized patients receiving the infusion (2.5 mg vs. 0.6 mg, *p* < 0.001), and the duration of infusion was longer (21.0 h vs. 6.9 h, *p* < 0.001). Adverse events related to flumazenil were significantly more common in patients receiving the infusion than in those not receiving it (44.5% vs. 21.3%, *p* < 0.001). The most common adverse event was aggressiveness or agitation, which occurred in 31.2% of patients receiving flumazenil infusion versus 6.6% of those without infusion (*p* < 0.001). Patients receiving flumazenil infusion had longer hospital stays (median 2.0 vs. 1.0 days, *p* = 0.019) and higher hospitalization rates (59.5% vs. 46.2%, *p* = 0.014) (Table 2).

**Figure 2:**
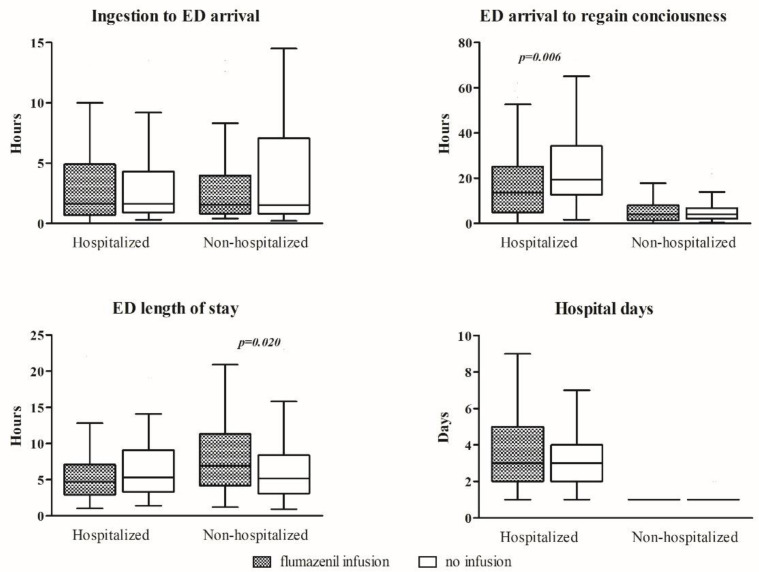
Box Plot of Time to Recovery of Consciousness According to Continuous Flumazenil Infusion and Hospitalization Status.

Figure 2 illustrates the relationship between continuous flumazenil infusion and the time to regain consciousness in patients with benzodiazepine poisoning, stratified by hospitalization status. Among hospitalized patients, those who received flumazenil infusion regained consciousness significantly faster than those who did not (median ED arrival to regain consciousness: 13.7 [4.9–24.8] h vs. 19.4 [12.8–33.8] h, *p* = 0.006). Similarly, the total time from ingestion to consciousness recovery was shorter in the infusion group (18.7 [8.2–31.7] h vs. 23.6 [15.5–36.4] h, *p* = 0.024). However, despite the more rapid recovery of consciousness, there was no significant difference in the total length of hospital stay between the infusion and noninfusion groups (3.0 [2.0–5.0] days vs. 3.0 [2.0–4.0] days, *p* = 0.466).

In contrast, among nonhospitalized patients, flumazenil infusion did not shorten the time to regain consciousness compared with those who did not receive the infusion (ED arrival to regain consciousness: 4.1 [1.4–7.9] h vs. 4.1 [2.0–6.7] h, *p* = 0.657; ingestion to regain consciousness: 7.0 [3.7–11.5] h vs. 7.7 [4.5–13.2] h, *p* = 0.342). Notably, the emergency department length of stay was significantly longer in the flumazenil infusion group (6.9 [4.2–11.3] h) than in the noninfusion group (5.1 [3.1–8.2] h, *p* = 0.02), whereas the total length of hospital stay remained the same (median 1.0 days for both groups, *p* = 0.252) (Figure 2).

### 3.3. Time to Regain Consciousness Analysis

The study population was stratified into quartiles on the basis of the time to regain consciousness from the time of arrival at the emergency department. Patients in the fourth quartile (longest time to consciousness, 19.2~111.6 h) demonstrated the most severe clinical presentation, with lower GCS scores on arrival (median 11.5 vs. 13.0 in the first quartile, *p* = 0.002). These patients required higher total flumazenil doses (median 2.5 mg vs. 0.6 mg in the first quartile, *p* < 0.001) and longer infusion durations (24.0 h vs. 4.9 h, *p* < 0.001).

Critical care interventions increased significantly with increasing time to consciousness recovery. The intubation rates increased from 4.3% in the first quartile to 21.7% in the fourth quartile (*p* = 0.007). Similarly, inotropic support requirements increased from 2.2% to 16.3% across quartiles (*p* = 0.001). Pneumonia complications were most common in the fourth quartile (25.0% vs. 5.4% in the first quartile, *p* = 0.002).

Hospitalization rates were strongly correlated with delayed consciousness recovery, ranging from 25.0% in the first quartile to 95.7% in the fourth quartile (*p* < 0.001). Similarly, hospital length of stay increased from 1.0 days in the first two quartiles to 4.0 days in the fourth quartile (*p* < 0.001) (Table 3).

### 3.4. Factors Associated with Time to Consciousness Recovery

Multiple linear regression analysis revealed several independent factors associated with prolonged time to regain consciousness. A lower GCS score at the time of arrival to the emergency department was significantly associated with delayed recovery (B = −1.592, 95% CI: −2.19 to −1.00, *p* < 0.001). A longer interval from ingestion to arrival at the emergency department was also correlated with delayed consciousness recovery (B = 0.255, 95% CI: 0.01–0.50, *p* = 0.038). Higher ingestion doses measured in defined daily dose units were associated with prolonged recovery times (B = 0.099, 95% CI: 0.01–0.19, *p* = 0.038).

Specifically, the GCS score on arrival remained the strongest predictor of delayed consciousness recovery (B = −1.063, 95% CI: −1.96 to −0.17, *p* = 0.020). Flumazenil infusion tended to be associated with prolonged recovery time in hospitalized patients, although this difference did not reach statistical significance (B = 5.406, 95% CI: −0.6311.45, *p* = 0.079) (Table 4).

## 4. Discussion

Unlike previous studies, this research clearly demonstrated in a real-world clinical setting that continuous flumazenil infusion significantly shortens the time to recovery of consciousness in severely affected hospitalized patients with benzodiazepine poisoning. At the same time, the study highlights a notable increase in adverse events and the limited impact on overall clinical outcomes and, most importantly, provides concrete evidence for the necessity of individualized, patient-tailored therapy. This distinction underscores the need for careful patient selection and the development of clear protocols to optimize both the efficacy and safety of flumazenil administration in clinical practice.

Our study demonstrated that, among hospitalized patients with benzodiazepine poisoning, flumazenil continuous infusion was associated with a significantly shorter time to regain consciousness than was not receiving the infusion. This finding is notable, as previous studies have offered mixed results regarding the impact of flumazenil on the duration of central nerve system (CNS) depression. Højer et al. demonstrated that continuous flumazenil infusion (0.5 mg/h) in patients with severe benzodiazepine overdose who initially responded to a bolus dose could maintain arousal and prevent relapse into coma, as measured by the Glasgow Coma Scale (GCS) [13]. Similarly, other reports have shown that continuous infusion can be effective in sustaining consciousness in cases where single bolus administration leads to only transient improvement [14,15,16]. However, many earlier studies focused on the maintenance of arousal or prevention of resedation rather than on a statistically significant reduction in overall time to recovery of consciousness. Our data provide additional evidence that, in real-world clinical practice, continuous flumazenil infusion can expedite the recovery of consciousness in selected severely affected patients. This effect, however, was not observed in nonhospitalized patients, where flumazenil infusion did not reduce the time to recovery and was associated with a longer emergency department (ED) length of stay. These findings suggest that the benefit of flumazenil in accelerating awakening may be limited to a subset of patients with more severe presentations who require hospitalization.

Despite the observed benefit in consciousness recovery among hospitalized patients, flumazenil infusion was associated with a significantly higher incidence of adverse events, particularly aggressiveness or agitation, in both hospitalized and nonhospitalized patients. Moreover, it is important to consider the complex pharmacodynamic interactions of flumazenil at the benzodiazepine receptor site. Beyond its established role as a competitive antagonist, flumazenil may exhibit competitive dualism-type interactions, whereby it can differentially modulate receptor activity depending on endogenous ligand presence and receptor subtypes. Such nuanced interactions may result in variable clinical effects in different patients, potentially influencing both therapeutic efficacy and adverse event profiles. These receptor-level dynamics underscore the complexity of interpreting flumazenil’s impact on consciousness recovery in benzodiazepine poisoning and highlight the need for individualized treatment strategies and careful patient monitoring [11,17]. In our study, the most frequent adverse reaction was aggressiveness or agitation, which occurred in 38.8% of hospitalized and 20.0% of nonhospitalized patients who received flumazenil, whereas it occurred in 14.3% and 0% of those who did not receive the infusion, respectively. Other adverse events, including tachycardia and pneumonia, did not significantly differ between the groups, and no seizures were observed in our cohort. These results are consistent with those of previous meta-analyses and clinical studies, which reported that flumazenil increases the risk of adverse events, particularly agitation and convulsions, compared with placebo [18]. Moreover, several patients in both groups experienced resedation after cessation of flumazenil, highlighting the need for careful monitoring and individualized dosing strategies. A systematic review by Penninga et al. demonstrated that adverse events were nearly three times more common in patients treated with flumazenil (risk ratio: 2.85), and serious adverse events—including seizures and cardiac arrhythmias—were also significantly more frequent (risk ratio: 3.81) [19]. Korean data similarly identified agitation (13.5%) as the most frequent side effect, with rare but serious events such as seizures and arrhythmias also reported [20]. Importantly, the overall mortality associated with flumazenil use remains low, and no deaths were observed during the blinded phases of the randomized trials. Notably, although flumazenil infusion led to a more rapid recovery of consciousness in hospitalized patients, this did not translate into a reduction in the total length of hospital stay. In nonhospitalized patients, flumazenil infusion was, in fact, associated with a longer ED length of stay. These findings underscore the clinical reality that the increased risk of adverse events may offset the benefit of faster awakening and that flumazenil does not necessarily improve broader clinical outcomes such as hospital throughput or resource utilization.

Our study revealed distinct clinical implications of continuous flumazenil infusion depending on hospitalization status. Compared with not receiving the infusion, flumazenil infusion was associated with a significantly shorter time to regain consciousness in hospitalized patients. However, this more rapid recovery did not translate into a reduction in the total length of hospital stay. In contrast, among nonhospitalized patients, flumazenil infusion did not shorten the time to regain consciousness and was instead associated with a longer emergency department length of stay. A key observation from our study is the lack of standardized protocols for flumazenil administration, which may have contributed to variable outcomes. The absence of clear criteria for patient selection and dosing has likely led to unnecessary or inappropriate use in some cases, as reflected by the lack of benefit and even prolonged ED stays in mild cases. Our findings emphasize the need for evidence-based protocols that can help clinicians identify patients who are most likely to benefit from flumazenil while minimizing the risk of adverse events. Furthermore, the high rate of complications observed in our cohort underscores the importance of close monitoring and individualized, tailored therapy on the basis of patient-specific risk factors and clinical indicators. This approach aligns with current international guidelines, which advocate for a careful risk–benefit assessment prior to flumazenil administration. In particular, patients with chronic benzodiazepine use, co-ingestion of proconvulsive agents, or underlying seizure disorders are at increased risk of adverse events and should be excluded from flumazenil therapy unless the anticipated benefit clearly outweighs the risks [7,21]. Our data suggest that, in the absence of clear protocols, flumazenil may be overused or misapplied, especially in less severe cases, leading to unnecessary exposure to adverse effects and inefficient use of health care resources. Establishing clear, evidence-based criteria for flumazenil administration and incorporating monitoring parameters for adverse events are essential for optimizing its use in clinical practice.

Multiple regression analysis in our study revealed that lower GCS scores at ED arrival, longer times from ingestion to ED presentation, and higher benzodiazepine doses (based on DDD) as independent predictors of delayed recovery of consciousness. These findings are consistent with prior research, which has similarly highlighted the severity of CNS depression, ingested dose, and delayed presentation as key determinants of prolonged recovery in patients with benzodiazepine poisoning. For example, Isbister et al. and others reported that patients presenting with lower initial GCS scores or higher ingested doses are at increased risk for prolonged CNS depression and require more intensive monitoring and supportive care [22,23]. Our results further suggest that the interplay between these factors and flumazenil efficacy may be more nuanced than previously appreciated, supporting the need for individualized treatment strategies. Notably, several predictors that were significantly associated with delayed recovery of consciousness in the overall cohort—such as lower GCS at presentation, higher ingested dose, and delayed ED arrival—did not retain statistical significance when analyzed within the hospitalized patient subgroup. This discrepancy may be attributed to several factors. First, the hospitalized population likely represents a more clinically homogeneous group of patients with greater severity, reducing variability across key clinical parameters. Second, confounding factors such as comorbid conditions and mixed drug ingestions may dilute the independent effects of these variables. Third, standardized inpatient monitoring and treatment—including vigilant supportive care and the potential use of flumazenil—may attenuate the impact of initial clinical features on the recovery trajectory. Fourth, the reduced sample size in the hospitalized subgroup may have limited the statistical power to detect significant associations. Finally, clinician-driven interventions, such as the titration of flumazenil or other sedatives, may have influenced outcomes in ways not fully captured by the measured variables. These considerations highlight the complexity of interpreting prognostic factors within more severe patient subgroups and emphasize the need for cautious interpretation. Future multicenter studies with larger sample sizes and stratified analyses are warranted to clarify the role of these variables across different severity levels of benzodiazepine poisoning. Notably, while flumazenil may expedite recovery in patients with severe presentations, its benefit is less apparent in those with milder intoxication or delayed presentation, where supportive care alone is often sufficient for full recovery.

In addition to clinical indications, the decision to hospitalize patients following a suicide attempt is often influenced by legal and social factors, which vary by country. For example, in South Korea, psychiatric admission after self-harm is frequently determined by considerations of family support, current risk of harm to self or others, and physician judgment, as guided by national guidelines and mental health law. In contrast, in countries such as the United States or those in Europe, legal mandates for involuntary psychiatric admission, specific suicide risk assessment tools, and broader access to community mental health services can play a critical role in determining whether hospitalization occurs. This diversity in hospitalization criteria—including legal, social, and cultural factors—impacts patient management, resource utilization, and outcome comparisons across international cohorts. In the present study, some admissions were based not only on clinical severity but also on psychosocial vulnerability or legal concerns. Future research and inter-study comparisons should consider such contextual differences in order to accurately interpret treatment indications and outcomes [24,25].

This study has several limitations. As a single-center retrospective analysis, there is potential for selection bias and limited generalizability to other settings. The reliance on medical records may have resulted in incomplete or inaccurate data, particularly regarding the amount and timing of benzodiazepine ingestion and the presence of coingestants. The decision to administer flumazenil was not standardized and may have been influenced by clinical judgment, introducing indication bias. Additionally, the retrospective design may have led to underreporting mild or transient adverse events, and long-term outcomes after discharge could not be assessed. The impact of emerging designer benzodiazepines on flumazenil efficacy and safety also remains unclear. Future prospective, multicenter studies are warranted to validate these findings and inform the development of standardized, evidence-based protocols for flumazenil use in benzodiazepine poisoning.

## 5. Conclusions

In conclusion, our study demonstrated that continuous flumazenil infusion can expedite the recovery of consciousness in selected severely affected hospitalized patients with benzodiazepine poisoning. However, this benefit does not extend to improvements in overall hospital or ED length of stay, likely due to a significantly increased risk of adverse events. These findings highlight the need for careful patient selection, individualized risk assessment, and the development of clear, evidence-based protocols for flumazenil administration in the management of benzodiazepine overdose. Ultimately, flumazenil should be reserved for carefully selected patients, and its use should be guided by both clinical judgment and robust evidence.

## Figures and Tables

**Table 1:** Baseline Characteristics According to Hospitalization Status.

	Total Cohort (*n* = 370)	Hospitalized (*n* = 194)	Non-Hospitalized (*n* = 176)	*p*
Male	104 (28.1%)	55 (28.4%)	49 (27.8%)	1.000
Age	46.0 [29.0; 61.0]	49.0 [28.0; 66.0]	45.0 [29.5; 58.5]	0.158
past NP history	224 (60.5%)	127 (65.5%)	97 (55.1%)	0.054
Depression	176 (47.6%)	99 (51.0%)	77 (43.8%)	0.195
Psychotic disorder	6 (1.6%)	3 (1.5%)	3 (1.7%)	1.000
Other	91 (24.6%)	52 (26.8%)	39 (22.2%)	0.360
Employed	69 (18.6%)	40 (20.6%)	29 (16.5%)	0.375
Live with family	176 (47.6%)	113 (58.2%)	63 (35.8%)	<0.001
Drug related				
Dose (DDD, units)	11.1 [5.8; 21.0]	13.1 [7.0; 28.0]	10.0 [4.0; 20.0]	0.004
co-injestion drug	150 (40.5%)	84 (43.3%)	66 (37.5%)	0.304
alcohol co-injestion	114 (30.8%)	55 (28.4%)	59 (33.5%)	0.335
Reattempt	111 (30.0%)	67 (34.5%)	44 (25.0%)	0.059
injestion to ED arrival	1.5 [0.8; 5.1]	1.6 [0.8; 4.6]	1.5 [0.8; 5.5]	0.856
at ED arrival				
GCS	13.0 [9.0; 13.0]	11.0 [8.0; 13.0]	13.0 [12.0; 13.0]	<0.001
Severity of conciousness				<0.001
GCS ≤ 8	84 (22.7%)	62 (32.0%)	22 (12.5%)	
GCS ≥ 9 and ≤13	267 (72.2%)	122 (62.9%)	145 (82.4%)	
GCS ≥ 14	19 (5.1%)	10 (5.2%)	9 (5.1%)	
Systolic blood pressure	113.0 [101.0; 129.0]	110.0 [98.5; 128.0]	116.0 [104.5; 129.0]	0.016
Diastolic blood pressure	73.0 [63.0; 84.0]	71.0 [62.0; 83.0]	74.5 [64.0; 85.0]	0.126
Heart rate	83.0 [72.0; 96.0]	82.0 [72.0; 94.0]	83.5 [74.0; 96.5]	0.607
Respiratory rate	20.0 [18.0; 20.0]	20.0 [18.0; 20.0]	20.0 [18.0; 20.0]	0.872
Temperature	36.5 [36.1; 36.9]	36.5 [36.0; 36.9]	36.5 [36.2; 36.8]	0.491
PH	7.38 [7.35; 7.41]	7.37 [7.33; 7.40]	7.39 [7.36; 7.41]	<0.001
PaCO_2_	40 [37; 44]	41 [37; 45]	39 [36; 42]	0.005
PaO_2_	82 [68; 93]	82 [69; 94]	81 [65; 92]	0.305
HCO_3−_	23.1 [21.3; 24.9]	23.1 [21.2; 24.9]	23.1 [21.6; 24.9]	0.716
Saturation	95.9 [93.4; 97.1]	95.8 [93.5; 97.2]	96.0 [92.4; 97.0]	0.704
ED management				
Charcoal	64 (17.3%)	33 (17.0%)	31 (17.6%)	0.988
Intubation	50 (13.5%)	38 (19.6%)	12 (6.8%)	0.001
Inotropics	33 (8.9%)	28 (14.4%)	5 (2.8%)	<0.001
Flumazenil	289 (78.1%)	148 (76.3%)	141 (80.1%)	0.374
Continuous infusion	173 (46.8%)	103 (53.1%)	70 (39.8%)	0.014
Dose, mg	1.0 [0.3; 3.0]	2.5 [0.5; 8.0]	0.6 [0.3; 1.5]	<0.001
Duration, hours	13.0 [7.2; 24.0]	21.0 [12.5; 26.0]	6.9 [4.2; 11.3]	<0.001

Values are means ± standard deviation or median (interquartile range) or numbers and percentages; NP, Neuropsychiatry; DDD, defined daily dose; ED, Emergency department; GCS, Glasgow Coma Scale.

**Table 2:** Clinical Outcomes According to Flumazenil Continuous Infusion.

	Flumazenil Infusion (*n* = 173)	No Infusion (*n* = 197)	*p*-Value
Time interval			
injestion to ED arrival	1.6 [0.8; 4.5]	1.5 [0.8; 5.8]	0.449
injestion to regain consciousness	11.2 [5.7; 23.1]	14.0 [5.9; 25.5]	0.305
ED arrival to regain consciousness	8.0 [2.8; 18.4]	8.4 [3.4; 19.4]	0.375
Adverse events related flumazenil	77 (44.5%)	42 (21.3%)	<0.001
Seizure	0 (0.0%)	1 (0.5%)	1.000
Tachycardia	34 (19.7%)	29 (14.7%)	0.262
Aggressiveness or agitation	54 (31.2%)	13 (6.6%)	<0.001
Pneumonia	28 (16.2%)	24 (12.2%)	0.339
Intubation required	24 (13.9%)	26 (13.2%)	0.970
Inotropics	13 (7.5%)	20 (10.2%)	0.481
ED length of stay	5.7 [3.5; 8.6]	5.2 [3.2; 8.9]	0.610
Hospital days	2.0 [1.0; 3.0]	1.0 [1.0; 3.0]	0.019
Hospitalized	103 (59.5%)	91 (46.2%)	0.014
Mortality	2 (1.2%)	0 (0.0%)	0.422

Values are means ± standard deviation or median (interquartile range) or numbers and percentages; ED, Emergency department.

**Table 3:** Associated Factors and Clinical Outcomes According to Quartiles of Time to Regain Consciousness from ED arrival.

	Q1 (0.1~3.1)	Q2 (3.2~8.2)	Q3 (8.3~19.1)	Q4 (19.2~111.6)	*p*
	(*n* = 92)	(*n* = 93)	(*n* = 93)	(*n* = 92)	
Time to Regain Consciousness, mean	1.4	5.3	12.8	37.5	
male	28 (30.4%)	20 (21.5%)	28 (30.1%)	28 (30.4%)	0.443
Age	48.0 [29.0; 59.5]	45.0 [30.0; 61.0]	43.0 [30.0; 57.0]	50.0 [27.0; 72.0]	0.624
Injestion dose, DDD	10.0 [5.5; 20.0]	13.0 [6.0; 25.0]	10.0 [4.4; 20.0]	14.0 [8.0; 30.0]	0.072
injestion to ED arrival	2.0 [0.9; 5.1]	1.4 [0.7; 4.3]	1.4 [0.9; 4.5]	1.7 [0.8; 7.4]	0.316
Co-ingestion drug	39 (42.4%)	30 (32.3%)	36 (38.7%)	45 (48.9%)	0.134
Co-injestion alcohol	30 (32.6%)	29 (31.2%)	26 (28.0%)	29 (31.5%)	0.914
GCS on ED arrival	13.0 [12.0; 13.0]	13.0 [10.0; 13.0]	12.0 [8.0; 13.0]	11.5 [8.0; 13.0]	0.002
flumazenil infusion	48 (52.2%)	40 (43.0%)	44 (47.3%)	41 (44.6%)	0.615
total dose, mg	0.6 [0.3; 1.5]	0.9 [0.3; 1.8]	2.2 [0.3; 3.8]	2.5 [0.3; 10.2]	<0.001
total duration, hours	4.9 [3.2; 12.5]	9.1 [7.1; 12.2]	15.7 [13.0; 24.0]	24.0 [17.0; 30.0]	<0.001
Intubation	4 (4.3%)	12 (12.9%)	14 (15.1%)	20 (21.7%)	0.007
Inostorpics	2 (2.2%)	4 (4.3%)	12 (12.9%)	15 (16.3%)	0.001
APACHE_II	6.0 [5.0; 10.0]	7.0 [4.5; 11.5]	9.0 [5.5; 13.0]	8.0 [5.0; 14.0]	0.505
Pneumonia	5 (5.4%)	13 (14.0%)	11 (11.8%)	23 (25.0%)	0.002
PH	7.39 [7.36; 7.41]	7.39 [7.36; 7.41]	7.37 [7.33; 7.40]	7.38 [7.33; 7.41]	0.008
PaCO_2_	40 [37; 42]	9 [36; 42]	40 [37; 44]	41 [37; 45]	0.229
PaO_2_	82 [68; 91]	81 [70; 95]	80 [65; 93]	82 [70; 96]	0.739
HCO_3_^−^	23.5 [21.9; 25.1]	23.1 [21.5; 24.6]	22.5 [21.1; 24.4]	23.1 [21.3; 25.0]	0.358
Saturation	96.0 [93.1; 97.0]	95.8 [93.8; 97.2]	95.7 [91.4; 96.9]	95.9 [93.5; 97.2]	0.781
Glucose, mg/dl	104.0 [95.0; 120.5]	103.0 [92.0; 123.0]	107.0 [96.0; 126.0]	112.5 [100.5; 134.5]	0.047
Outcomes					
Adverse events related flumazenil	24 (26.1%)	24 (25.8%)	34 (36.6%)	37 (40.2%)	0.077
Seizure	0 (0.0%)	0 (0.0%)	0 (0.0%)	1 (1.1%)	0.387
Tachycardia	13 (14.1%)	14 (15.1%)	20 (21.5%)	16 (17.4%)	0.546
Aggressiveness or agitation	12 (13.0%)	12 (12.9%)	16 (17.2%)	27 (29.3%)	0.011
Hospitalized	23 (25.0%)	26 (28.0%)	57 (61.3%)	88 (95.7%)	<0.001
Mortality	0 (0.0%)	0 (0.0%)	0 (0.0%)	2 (2.2%)	0.108
ED length of stay	3.2 [2.2; 4.9]	6.0 [4.7; 7.6]	9.1 [4.4; 12.4]	5.8 [3.4; 9.1]	<0.001
Hospital days	1.0 [1.0; 1.0]	1.0 [1.0; 1.0]	2.0 [1.0; 3.0]	4.0 [2.0; 5.0]	<0.001

Values are means ± standard deviation or median (interquartile range) or numbers and percentages; DDD, defined daily dose; ED, Emergency department; GCS, Glasgow Coma Scale; APACHE II, Acute physiology and chronic health evaluation II.

**Table 4:** Multiple linear regression analysis between associated factors and time interval from ED arrival to regain consciousness.

	Total Cohort			Hospitalized		
	B	95% CI	*p*	B	95% CI	*p*
male	−0.009	−3.90~3.88	0.996	−0.171	−6.71~6.37	0.959
Age	0.046	−0.04~0.13	0.314	0.044	−0.10~0.19	0.545
GCS on ED arrival	−1.592	−2.19~−1.00	<0.001	−1.063	−1.96~−0.17	0.020
injestion to ED arrival	0.255	0.01~0.50	0.038	0.354	−0.03~0.74	0.074
Injestion dose, DDD (unit)	0.099	0.01~0.19	0.038	0.071	−0.06~0.21	0.303
Co-ingestion drug	−3.553	−7.13~0.02	0.051	−3.604	−9.55~2.35	0.234
Co-ingestion alcohol	2.071	−1.59~5.73	0.266	0.356	−5.96~6.67	0.911
Continuous flumazenil infusion	1.555	−1.89~5.00	0.375	5.406	−0.63~11.45	0.079

DDD, defined daily dose; ED, Emergency department; GCS, Glasgow Coma Scale.

## Data Availability

Due to privacy restrictions all data are stored at the researcher’s institution. Qualified researchers will be able to gain access via application at the corresponding author.
